# Spinal Anaesthesia Using Hypobaric Drugs: A Review of Current Evidence

**DOI:** 10.7759/cureus.56069

**Published:** 2024-03-13

**Authors:** Naresh Paliwal, Minal V Kokate, Nandini A Deshpande, Imran Ahmed Khan

**Affiliations:** 1 Anesthesiology, Dr. Panjabrao Deshmukh Memorial Medical College, Amravati, IND; 2 Community Medicine, Baba Raghav Das Medical College, Gorakhpur, IND

**Keywords:** spinal anesthesia, ropivacaine, local anesthesia, hypobaric, bupivacaine

## Abstract

Spinal anesthesia is one of the most widely used techniques in modern anesthesia practice. It involves the injection of local anesthetic drugs into the cerebrospinal fluid (CSF) within the subarachnoid space. The choice of drug, its concentration, and baricity play a crucial role in determining the characteristics of the spinal block and has evolved over the years with continuous advancements in drug formulations and administration methods. Spinal anesthesia with hypobaric drugs represents a valuable technique in the armamentarium of anesthesiologists, offering distinct advantages in terms of targeted action, reduced systemic toxicity, and enhanced hemodynamic stability. This review aims to scan the characteristics of hypobaric drugs, factors influencing their spread within the spinal canal, challenges associated with their use, clinical applications in various surgical scenarios, and potential implications for patient outcomes and healthcare practice. PubMed and Google Scholar databases were searched for relevant articles and a total of 23 relevant articles were selected for the review based on inclusion and exclusion criteria. Hypobaric drugs have many advantages in high-risk morbidly ill patients for some select surgical procedures and daycare surgeries. The concentration and volume of hypobaric drugs need to be selected according to the extensiveness of the surgery and the desired block can be achieved by giving spinal injection in specific positions. The dynamic field of anesthesiology encompasses the integration of emerging technologies and evidence-based practices, which will contribute to further refining the safety and efficacy of spinal anesthesia with hypobaric drugs.

## Introduction and background

Spinal anesthesia is one of the most widely used techniques in modern anesthesia practice [1], and is a technique that is easy to learn and master. Also known as subarachnoid block, spinal anesthesia involves the injection of local anesthetic drugs into the cerebrospinal fluid (CSF) within the subarachnoid space. This results in a reversible loss of sensation and motor function below the level of injection, providing effective anesthesia for surgeries involving the lower abdomen, pelvis, and lower limbs [2].

The choice of drug, its concentration, and baricity play a crucial role in determining the characteristics of the spinal block. Spinal anesthesia is most commonly performed below L1 using hyperbaric drugs only. This may be because of ignorance about modern anatomy and other drugs like isobaric and hypobaric. It has evolved over the years with continuous advancements in drug formulations and administration methods [3,4].

This review comprehensively explores the current evidence regarding the use of hypobaric drugs in spinal anesthesia. Specifically, the review aims to scan the characteristics of hypobaric drugs, factors influencing their spread within the spinal canal, challenges associated with their use, clinical applications in various surgical scenarios, and potential implications for patient outcomes and healthcare practice. This will help in evaluating the safety, efficacy, and practical considerations during spinal anesthesia with hypobaric drugs, with the ultimate goal of informing anesthesia practitioners and guiding future research in this field.

## Review

Methods

The present review was conducted to compile current evidence regarding the use of hypobaric drugs in spinal anesthesia. We searched PubMed and Google Scholar databases for relevant articles published from 2014 up to December 2023 using the keywords spinal anesthesia, subarachnoid block, and hypobaric. We included all articles that had the above keywords in the title and/or abstract. Review articles, in vitro studies, articles not available in English, and studies on animals were excluded.

Results and discussion

The initial search yielded a total of 38 publications (32 from PubMed and six from Google Scholar). After removing duplicates, 33 articles remained. Three articles were removed as they were systematic reviews and metanalyses, three were animal studies, two were in vitro studies, one article did not have the full text available in the English language, and free text was not available for one study. Finally, 23 relevant articles were selected for the review. The included articles are given in Table 1.

**Table 1:** Brief description of included articles CSA, continuous spinal anesthesia; CSEA, combines spinal epidural anesthesia; FICB, fascia iliaca compartment block; GA, general anesthesia; HAS, hypobaric spinal anesthesia; HUSA, hypobaric unilateral spinal anesthesia; MFS, marfan syndrome; SSSA, single-shot spinal anesthesia; STSA, segmental thoracic spinal anesthesia; THR, total hip replacement

S. No.	Authors/Study	Study design	Conclusion
1	Quan et al., 2014 [5]	Original article	The combination of hyperbaric and hypobaric ropivacaine produced adequate anesthetic effects and a more stable hemodynamic profile than either medication administered alone.
2	Zhu et al., 2014 [6]	Randomized prospective study	Unilateral spinal anesthesia can provide limited sensory and motor block, minimize the incidence of hypotension, and prevent the stress responses undergoing THR resulting in an optimal anesthesia procedure for geriatric patients by rapid subarachnoid injection of small doses of bupivacaine.
3	de la Rica et al., 2014 [7]	Letter to Editor	Hypobaric spinal anesthesia is a safe and useful technique for paraplegic patients undergoing surgery in a jack-knife position.
4	Errando et al., 2014 [8]	Prospective, randomized, double-blinded study	Reducing the hypobaric bupivacaine dose to 3.75 mg in subarachnoid anesthesia for hip fracture repair surgery in elderly patients decreased intraoperative blood pressure, but in a considerable number of patients, rescue anesthesia was needed.
5	Cuchillo-Sastriques et al., 2014 [9]	Case report	Hemodynamic stability and analgesia were satisfactory in all cases with a hypobaric technique.
6	Quan et al., 2015 [10]	Prospective, double-blind, randomized, controlled study	Combined use of hyperbaric and hypobaric ropivacaine significantly decreased the incidences of complications in spinal anesthesia for cesarean section
7	Zhao, 2015 [11]	Letter To the Editor	Query about the density of ropivacaine
8	Cantürk, 2016 [12]	Letters to the Editor	The resultant mixed solution (ropivacaine and water) was hypobaric
9	Vergari A et al (2016) [13]	Original article	Isobaric levobupivacaine has a shorter onset time for sensory block and delays the regression of sensory and motor block on the nondependent side
10	Xu Z et al (2017) [14]	Original article	In comparison to the lateral postures, CSEA with hypobaric ropivacaine in the sitting position is more likely to elicit hypotension and an abnormally high block level.
11	Wang et al., 2017 [15]	Prospective, double-blinded, randomized dose-response trial	The hypobaric local anesthetic dose required for unilateral spinal anesthesia is low in geriatric patients undergoing hip replacement surgery
12	Kahloul et al., 2017 [16]	Prospective, randomized, double-blind study	In unilateral spinal anesthesia, 5mg of hypobaric bupivacaine is equally efficacious as 7.5mg, with less bilateralization and improved hemodynamic stability.
13	Biji et al., 2017 [17]	Prospective cohort study	Compared to near isobaric drugs, hypobaric solutions spread more in CSF with faster onset and higher upper sensory levels
14	Meuret et al., 2018 [18]	Prospective randomised open trial	HUSA provides better hemodynamic stability and safety than general anesthesia in elderly patients undergoing hip fracture surgery
15	Skjellerup, 2018 [19]	Case report	This case describes the successful administration of spinal anesthesia for cesarean section in a woman with MFS complicated by dural ectasia.
16	Armendáriz-Buil et al., 2019 [20]	Case report	HSA at low doses is an option in high abdominal wall surgery.
17	Aslan and Moraloğlu, 2020 [21]	Original article	The addition of fentanyl or high-dose morphine to bupivacaine improves the efficacy and duration of SSSA in the active phase of labor with fewer side effects.
18	Simonin et al., 2022 [22]	Prospective Randomised Open Trial	HUSA leads to fewer episodes of severe intraoperative hypotension compared to GA in an elderly population undergoing hip fracture surgery.
19	Vincenzi et al., 2022 [23]	Case report	STSA is a safe, reliable, and adequate anesthetic technique in surgeries involving the breast and axillary region, particularly in frail and elderly patients, representing a reasonable alternative to GA
20	Tang et al., 2022 [24]	Original article	FICB combined with HSA can successfully lower the incidence of early postoperative cognitive impairment in elderly patients with high-risk hip replacement
21	Kaabachi et al., 2022 [25]	Randomized controlled trial	Hypobaric bupivacaine may be used rather than isobaric bupivacaine for further preserving hemodynamics in CSA for hip fracture surgery in the elderly.
22	Vincenzi et al., 2023 [26]	Case series	Hypobaric opioid-free STSA appears to be a potential option for laparoscopic procedures, with minimum or no shoulder pain.
23	Kashanian et al., 2023 [27]	Retrospective chart review	Hypobaric lidocaine provided a significantly reduced mean ambulation and discharge times compared to either isobaric or hyperbaric bupivacaine, as well as a reduced discharge time compared to GA.

Before proceeding to hypobaric spinal anesthesia, it is essential to know a few important points about baricity and factors affecting the density of CSF, local anesthetic drugs, etc. Baricity is the ratio of the density of the local anesthetic solution to the density of CSF. Specifically, the baricity of a solution determines whether it will tend to float or sink in relation to CSF [28]. Density is defined as mass per unit volume of solution (g/lit) at a specific temperature. Density may be compared between different substances by calculating the specific gravity, which is the ratio of the density of a solution to the density of water. Because density varies with temperature, the baricity of a local anesthetic solution is conventionally defined at 37 degrees Celcius (^o^C). The density of CSF is 1.00059 g/lit [29].

**Figure 1:**
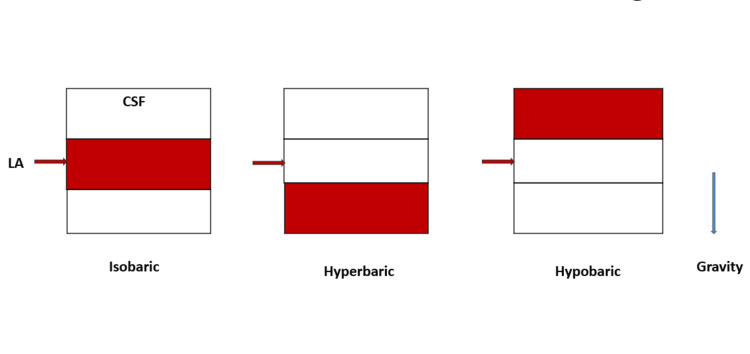
Distribution of local anesthetics in CSF according to baricity CSF, cerebrospinl fluid; LA, local anesthetic

Local anesthetic solutions that have the same density as CSF are termed isobaric, those that have a higher density than CSF are hyperbaric, and those with a density lower than CSF are hypobaric. Hypobaricity is defined as a solution with a density lower than three standard deviations below the mean density of cerebrospinal fluid [30]. Baricity is an important factor that influences the distribution of the anesthetic solution within the spinal canal [13,31]. The spread of hyperbaric solutions is more predictable with less inter-patient variability. For practical purposes, solutions with a baricity < 0.9990 are hypobaric and solutions with a baricity of ≥1.0015 are hyperbaric [17]. To make the drug hyperbaric to CSF it must be denser than CSF, the reverse is true for making a drug hypobaric to CSF. Dextrose is added to make a drug hyperbaric and distilled water (DW) is added to make it hypobaric [26]. Hyperbaric drugs spread to dependent regions of the spinal cord whereas hypobaric drugs will spread to non-dependant regions. Isobaric drugs are not influenced by gravity [5]. The spread of intrathecal drugs in the CSF is shown in Figure 1. Increasing the temperature of a drug decreases the density of the local anesthetic solution [32]. Plain bupivacaine 0.5%, for example, may be isobaric at 24^o^C but it is slightly hypobaric at 37^o^C [33,34].

Hypobaric drugs are not available; we need to prepare them from isobaric drugs by the addition of sterile DW. Commonly used drugs for making hypobaric in the order of potency are 0.5% bupivacaine > 0.5% levobupivacaine > ropivacaine 0.75%. Lignocaine 2% without preservatives is also used for short surgical procedures. But fear of cauda equina syndrome prevents its regular use [35]. Another method of formulating a hypobaric drug is to warm the isobaric drugs to 37^o^C; at this temperature, it can demonstrate hypobaric characteristics when used in sitting/lateral or prone positions [11,12]. The addition of opioids like fentanyl to isobaric drugs can make it a little hypobaric and patients may get higher levels if the spinal anesthesia is given in a sitting position and the patient is kept seated for some time [14,21,36]. The spread and selectivity of block achieved with hypobaric drugs are volume and concentration-dependent [6]. The lower the concentration of the drug used, the more selective (preferential sensory block) the block is achieved and the more the volume used, the more unpredictable the spread is [16]. So, the concentration and volume of hypobaric drugs need to be prepared and used according to surgical requirements.

The addition of 3 ml of sterile distilled water to 2 ml of 0.5% bupivacaine (10 mg) will make 0.2% solution (2 mg/ml) with a density of 0.99931. The addition of 3.5 ml of sterile distilled water to 1.5 ml of 0.5% bupivacaine will make 0.15% solution (1.5 mg/ml) with a density of 0.99815. The addition of 4 ml of sterile distilled water to 1 ml of 0.5% bupivacaine will make 0.1% (1 mg/ml) solution with a density of 0.99726. Similarly, the addition of 3.5 ml sterile distilled water to 1.5 ml (30 mg) of 2% lignocaine will make 0.6% (6 mg/ml) solution.

Challenges in Spinal Anesthesia Using Hypobaric Drugs

Hypobaric drugs need to be prepared so chances of contamination are there. There is less difference in density between hypobaric drugs and CSF as compared to that between hyperbaric drugs and CSF, so they are less predictable in terms of spread and levels of block achieved as compared to hyperbaric drugs [37]. Achieving a uniform block with hypobaric drugs poses difficulty due to the influence of patient position, drug concentration, and baricity. Adequate understanding and meticulous technique are essential to overcome these challenges. This necessitates careful titration and adjustment of drug dosage to achieve the desired block height. Patient positioning significantly affects the distribution of hypobaric spinal anesthesia. Changes in position during surgery may alter the spread of the drug, requiring vigilant monitoring and adjustments to maintain the desired block level [38-40]. Less muscle relaxation occurs when a low concentration of hypobaric drugs is used, so the surgeon needs to be accustomed to this less relaxation.

Uses of Hypobaric Drugs

Hypobaric drugs have very minimal hemodynamic fluctuations so can be used for high-risk cases of short to mid-duration surgeries [9,20]. Low doses of drugs used lead to preferential sensory blockade with minimal motor involvement and that is responsible for early recovery, ambulation, voiding, and discharge [27]. Hypobaric spinal anesthesia is associated with improved hemodynamic stability compared to hyperbaric solutions [19,41]. This is particularly beneficial in elderly patients or those with compromised cardiovascular function [23]. A randomized controlled study found that the combined use of hyperbaric and hypobaric ropivacaine significantly decreased the incidences of hypotension and complications in spinal anesthesia for cesarean section. [10] Spinal anesthesia using hypobaric drugs is commonly used for unilateral spinal anesthesia in lower limb surgeries, and anorectal surgeries in a prone Jack-knife position [15,18,22,24]. Apart from these common indications, hypobaric spinal can also be useful for producing posterior spinal hemi-anesthesia blocking only dorsal sensory roots when the spinal is given in the prone position [25]. This can be useful for procedures like endoscopic discectomy or other superficial surgeries on the back in the prone position [42]. Some lateral position superficial surgeries on thoracic and abdominal regions as well are possible like the lower limb surgeries. Another useful utility of hypobaric drugs is they can be used in combination with either hyperbaric or isobaric drugs to achieve the desired cephalic or caudal spread [10].

Unilateral Spinal Anesthesia Using Hypobaric Drugs

Unilateral spinal anesthesia is feasible with hypobaric drugs for lower limb orthopedic, plastic, and endovascular surgeries as well as some superficial thoracolumbar surgeries when the spinal is given in lateral position adjusting the tilt, level of injection, volume, and concentration of drug according to surgery [14,43]. For lower limb surgeries, spinal anesthesia needs to be given in a lateral position at lumber levels with a 10-15-degree head-down tilt. For lateral position surgeries, it is very useful as the operative and painful side is non-dependant and surgery can be continued in the same position, saving time. For other surgeries in the supine position, the patient needs to be kept in a lateral position after the spinal for 8-10 minutes. For surgeries at thoracic levels, spinal anesthesia can be given in a lateral position at lower thoracic levels with the operative side up and 10-15-degree head-up tilt. The patient needs to be kept in the same position for 8-10 minutes before being supine. For lower limb superficial surgeries like debridement etc., just 0.1-0.15% bupivacaine and a volume of 2-3 ml can be sufficient. For surgeries like bipolar arthroplasty or fracture femur etc., where some relaxation is needed, higher concentrations like 0.25 or even 0.375 % can be used for unilateral spinal in high-risk patients [8].

Anorectal Surgeries in Jack-Knife Position

The prone Jack-knife position is used for a variety of short-duration anorectal and plastic surgeries [7]. To facilitate performing the lumber puncture with the patient in the prone position, a pillow should be placed under the abdomen and pelvis to correct the lordosis and increase the interspinous space. In this position, the CSF appears spontaneously but if there is difficulty, the patient should be asked to cough or the CSF could be aspirated with a small syringe [44]. Keeping the patient in the Jack-knife position with a 10-15-degree head-down tilt and giving the spinal in the prone position at L2/3/4 space using around 3 ml of 0.15% bupivacaine is sufficient to provide surgical anesthesia for surgeries like pilonidal sinus, fistula, and piles. Relaxation of puborectalis muscle may not be adequate with this low concentration of drug, so deeper dissection of rectal lesions (high fistulas, polyps, etc.) may need the use of a higher concentration of hypobaric drugs. Additives like fentanyl can be used to increase the intensity of the block [45].

Posterior Spinal Hemianesthesia

It can be achieved by blocking only dorsal roots with hypobaric drugs by giving the spinal in the prone position. Several types of orthopedic surgeries (like endoscopic discectomy etc.) anorectal, plastic, and superficial surgeries of short to mid duration on the back can be done under this technique [46]. A dose of 5-7.5 mg of bupivacaine/levobupivacaine, that is around 3-5 ml of 0.15% hypobaric drug, is sufficient for surgeries of 60-90 minutes duration.

Hypobaric Drugs in Combination With Isobaric Drugs to Achieve the Desired Cephalic Spread

Low concentrations of hypobaric drug (0.1-0.15%) can be used in combination with an isobaric drug to achieve adequate cephalic spread, especially in cases like laparoscopic cholecystectomy, to prevent shoulder tip pain. For laparoscopic cholecystectomy, a little head-up tilt is used for surgery and that is beneficial for the cephalic spread of the hypobaric drug. It can be achieved by just 1-2 ml of 0.1% hypobaric drug followed by 1-1.5 ml of isobaric 0.5% drug in different syringes with spinal given at around T10 levels in a sitting position. Adequate selective block to lower cervical roots and effective surgical anesthesia till T3/4 can be achieved with this combination to avoid shoulder tip pain without any respiratory compromise [47].

Table 2 shows a SWOT (Strengths, Weaknesses, Opportunities, and Threats) analysis of using hypobaric drugs in spinal anesthesia. Hypobaric drugs in spinal anesthesia exhibit strengths such as precision, rapid onset, and fewer side effects. However, they are limited by a short duration of action and limited applicability. Opportunities lie in expanding indications beyond traditional spinal anesthesia and future scope in research. Nevertheless, threats include competition from standard therapies, regulatory challenges, and safety concerns that could impact adoption and routine usage, highlighting the need for continuous research and improvement.

**Table 2:** SWOT (Strength, Weakness, Opportunity, Threat) analysis of hypobaric drugs in spinal anesthesia

SWOT analysis			
Strength	Weakness	Opportunity	Threat
Precision, Rapid onset, Minimal systemic effects, Reduced complications	Patient variability, Limited duration, Skill-dependent, Limited applicability	Advancements in technology, Expanded indications, Collaborative research, Enhanced training programs	Regulatory challenges, Competition from alternative techniques, Cost considerations, Public perception

Limitations

We have searched only two databases namely PubMed and Google Scholar because of resource constraints and no funding for the project. Most other databases are paid and thus we did not have access to them. A narrative review, instead of a systematic review was done due to the same reasons. Although PubMed and Google Scholar searches are likely to cover most of the articles, there is still a possibility that a few articles might have been left out. We also limited our data research to the past 10 years to keep only updated and relevant data.

## Conclusions

Spinal anesthesia using hypobaric drugs represents a valuable technique in the armamentarium of anesthesiologists, offering distinct advantages in terms of targeted action, reduced systemic toxicity, and enhanced hemodynamic stability. The concentration and volume of hypobaric drug need to be selected according to the extensiveness of the surgery and the desired block can be achieved by giving spinal anesthesia in specific positions. While challenges such as inconsistent block characteristics and variable spread exist, ongoing research and technological innovations aim to address these limitations.
